# Impact of short-term discontinuation of ivermectin-based chemoprevention on onchocerciasis transmission in endemic settings with long history of mass drug administration

**DOI:** 10.1371/journal.pntd.0011250

**Published:** 2023-04-14

**Authors:** Arnauld Efon-Ekangouo, Hugues C. Nana-Djeunga, Narcisse Nzune-Toche, Raïssa Dongmo-Yemele, Jean Bopda, Viviane Ongbassomben, Laurentine Sumo, Anne Geiger, Thomas B. Nutman, Joseph Kamgno

**Affiliations:** 1 Higher Institute for Scientific and Medical Research (ISM), Yaoundé, Cameroon; 2 Department of Animal Biology and Physiology, Faculty of Science, University of Ebolowa, Ebolowa, Cameroon; 3 INTERTRYP, Institut de Recherche pour le Développement (IRD), University of Montpellier, Montpellier, France; 4 National Institute of Allergy and Infectious Diseases (NIAID), National Institutes of Health (NIH), Bethesda, Maryland, United States of America; 5 Department of Public Health, Faculty of Medicine and Biomedical Sciences, University of Yaoundé I, Yaoundé, Cameroon; Fundacao Oswaldo Cruz, BRAZIL

## Abstract

**Background:**

The control of onchocerciasis currently relies on annual distribution of single dose ivermectin. Because ivermectin has minimal effects on the adult parasite, mass drug administration (MDA) campaigns against onchocerciasis require at least 15 years of annual uninterrupted ivermectin distribution. Mathematical models have predicted that short-term disruption of MDA (as was seen during COVID-19) could impacted the microfilaridermia prevalence depending on the pre-control endemicity and the histories of treatment, requiring corrective measures (such as biannual MDA) to mitigate the effect on onchocerciasis elimination. Field evidence supporting this prediction, however, has yet to be gathered. This study aimed to assess the impact of ~2 years disruption of MDA on onchocerciasis transmission indicators.

**Methodology:**

A cross-sectional survey was carried out in 2021 in seven villages of Bafia and Ndikinimeki, two health districts located in the Centre Region, Cameroon, where MDA has been ongoing for two decades, but interrupted in 2020 as a response to the COVID-19 pandemic. Volunteers aged 5 years and above were enrolled for clinical and parasitological examinations for onchocerciasis. Data were compared with pre-COVID-19 prevalence and intensity of infection from the same communities to measure changes over time.

**Principal findings:**

A total of 504 volunteers (50.3% males), aged 5–99 years (Median: 38; IQR: 15–54) was enrolled in the two health districts. The overall prevalence of microfilaridermia in 2021 was similar in Ndikinimeki health district (12.4%; 95% CI: 9.7–15.6) and Bafia health district (15.1%; 95% CI: 11.1–19.8) (*p-value* = 0.16). Microfilaridermia prevalences were either similar between 2018 and 2021 in the communities of Ndikinimeki health district (19.3% vs 12.8% (*p* = 0.057) for Kiboum 1; and 23.7% vs 21.4% (*p* = 0.814) for Kiboum 2), or higher in 2019 compared to 2021 in the communities of Bafia health district (33.3% vs 20.0% (*p* = 0.035) for Biatsota). The mean microfilarial densities in these communities dropped from 5.89 (95% CI: 4.77–7.28) mf/ss to 2.4 (95% CI: 1.68–3.45) mf/ss (p-value < 0.0001), and from 4.81 (95% CI: 2.77–8.31) mf/ss to 4.13 (95% CI: 2.49–6.86) mf/ss (p-value < 0.02) in Bafia and Ndikinimeki health districts, respectively. Community Microfilarial Load (CMFL) dropped from 1.08–1.33 mf/ss in 2019 to 0.052–0.288 mf/ss in 2021 in Bafia health district while remaining stable in the Ndikinimeki health district.

**Conclusion/Significance:**

The continued decline in prevalence and CMFL observed ~2 years after MDA disruption is consistent with mathematical predictions (ONCHOSIM) and shows that additional efforts and resources are not needed to mitigate the effects of short-term MDA disruption in highly endemic settings prior to intervention with long treatment histories.

## Author summary

Onchocerciasis is a vector-borne parasitic infection associated with significant disabilities including permanent blindness. *Onchocerca volvulus* is endemic in 31 countries in Africa and Yemen where 220 million of people remain at risk. The control of onchocerciasis relies on annual or bi-annual mass administration of ivermectin. This approach has had a substantial impact on the burden of the disease and has led to interruption of the transmission of the infection in some areas. Because of the COVID-19 pandemic, many control programs, including MDA, were disrupted; mathematical models predicted that such disruption could affect the control efforts in some endemic settings (mainly in highly endemic areas prior to intervention and areas with short histories of MDA) and suggested mitigation measures such as increasing the frequency of MDA rounds. This study aimed to evaluate the impact of one missed round of ivermectin MDA on the burden of onchocerciasis and its endemicity in the Mbam valley in Cameroon. This study revealed that the decrease in parasitological indicators of the *O*. *volvulus* infection subsequent to the MDA disruption, was consistent with the mathematical predictions of ONCHOSIM model.

## Introduction

Onchocerciasis (river blindness) is a neglected tropical disease that remains endemic in 31 countries in Africa and in limited foci in two countries in Latin America and in one country in Asia (Yemen) [1]. The 2017 global burden of onchocerciasis was estimated to have 87 million being at risk of contracting the infection, of which about 20.9 million were infected and 1.15 million were blind [1]. Onchocerciasis is caused by *Onchocerca volvulus*, a parasitic nematode transmitted to humans through exposures to repeated bites of infected blackflies belonging to the genus *Simulium* which breed in fast-flowing rivers and streams. Adult *O*. *volvulus* are long-lived—lifespan of up to 17 years—typically found in subcutaneous nodules and from which female worms release thousands of microfilariae daily [2,3]. These microfilariae can be found in the skin and the eye where they drive pathological conditions such as lichenified onchodermatitis, papular dermatitis, skin depigmentation and/or atrophy, lymphadenopathy, keratitis, and iridocyclitis, the ultimate complication being blindness [4].

Efforts to control and eliminate onchocerciasis as a public health problem begun with vector control coordinated by the Onchocerciasis Control Program (OCP) launched by the World Health Organization in 1974 in eleven countries in West Africa [5]. In the 1980s, ivermectin (Mectizan)—a dihydro derivative of avermectin [6,7]—was used to sustain the efforts of OCP and became the mainstay of subsequent control programs including the African Program for Onchocerciasis Control (APOC) which ended in 2015 and replaced by the Expanded Special Project for Elimination of Neglected Tropical Diseases (ESPEN) [8]. The strategy used by APOC was the community-directed treatment with ivermectin (CDTI), a breakthrough strategy that enabled to improve geographic and therapeutic coverages [9,10]. In 2019, CDTI alone was used to provide ivermectin to more than 152.9 million people in Africa, representing more than 70% coverage of the 217.2 million population who require preventive chemotherapy for onchocerciasis in Africa region [8]. These mass drug administration (MDA) efforts relying on annual ivermectin distribution should be sustained for 15–20 years (and even more) [11]. since ivermectin is not adulticidal (this compound is primarily microfilaricidal) and the adult worms are long-lived.

The emergence of the SARS-CoV-2 global pandemic in 2020 [12] led to serious disruptions in public health services on a global scale [13,14]. These disruptions were particularly pronounced in low- and middle-income countries due to already under resourced healthcare systems [15]. In April 2020, the WHO issued a recommendation to postpone MDA and other interventions against NTDs in recognition of the potential risks of these activities to increase the spread of COVID-19 infection. This WHO recommendation was followed by a subsequent guidance issued in July 2020 providing a decision-making framework for implementation of mass treatment interventions, active case-finding campaigns and population-based surveys for neglected tropical diseases in the context of the COVID-19 pandemic. This two-step approach (risk-benefit assessment and precautionary measures) was progressively taken by NTD program managers and their supporting partners, and some countries quickly resumed activities, including preventive chemotherapy, in late 2020. However, this process was country-dependent, and many countries including Cameroon, resumed CDTI in 2021, that is a year later after WHO guidance has been issued. As a consequence, there were concerns raised about onchocerciasis-associated morbidity that would gradually increase towards the preintervention levels as a result of the MDA delay and that it would derail progress towards the 2021–2030 roadmap for NTDs [16]. Mathematical models (e.g. ONCHOSIM [17] and EPIONCHO [18,19] suggested that a delay of 1 or 2 years of MDA would drive increases of microfilarial prevalence and negatively impact elimination goals in settings with high pre-control microfilaridermia prevalence [20].

This study aimed at assessing the trends of epidemiological indicators after 20 months of disruption of the annual treatment regime of MDA with ivermectin for onchocerciasis control in two highly endemic health districts prior to intervention, with long preventive chemotherapy history.

## Methods

### Ethics statement

The protocol of this study was approved by the Centre Regional Ethics Committee for Human Health Research (CRERSH-Ce) (N° CE 1997/CRERSHC/2020). The study was conducted in accordance with the Declaration of Helsinki. Indeed, prior to inclusion process, participants were informed about the objectives of the study, the sampling procedures, the potential risks, the benefits and their right to freely withdraw their consent at any time. Participation was voluntary and all enrollees signed written informed consent (participants aged 18 years and above) or required parental consents (participants aged <18 years old). A unique identifier (barcode) was attributed to each enrollee for confidentiality purpose, and all data generated were treated anonymously.

### Study area

**Fig 1:**
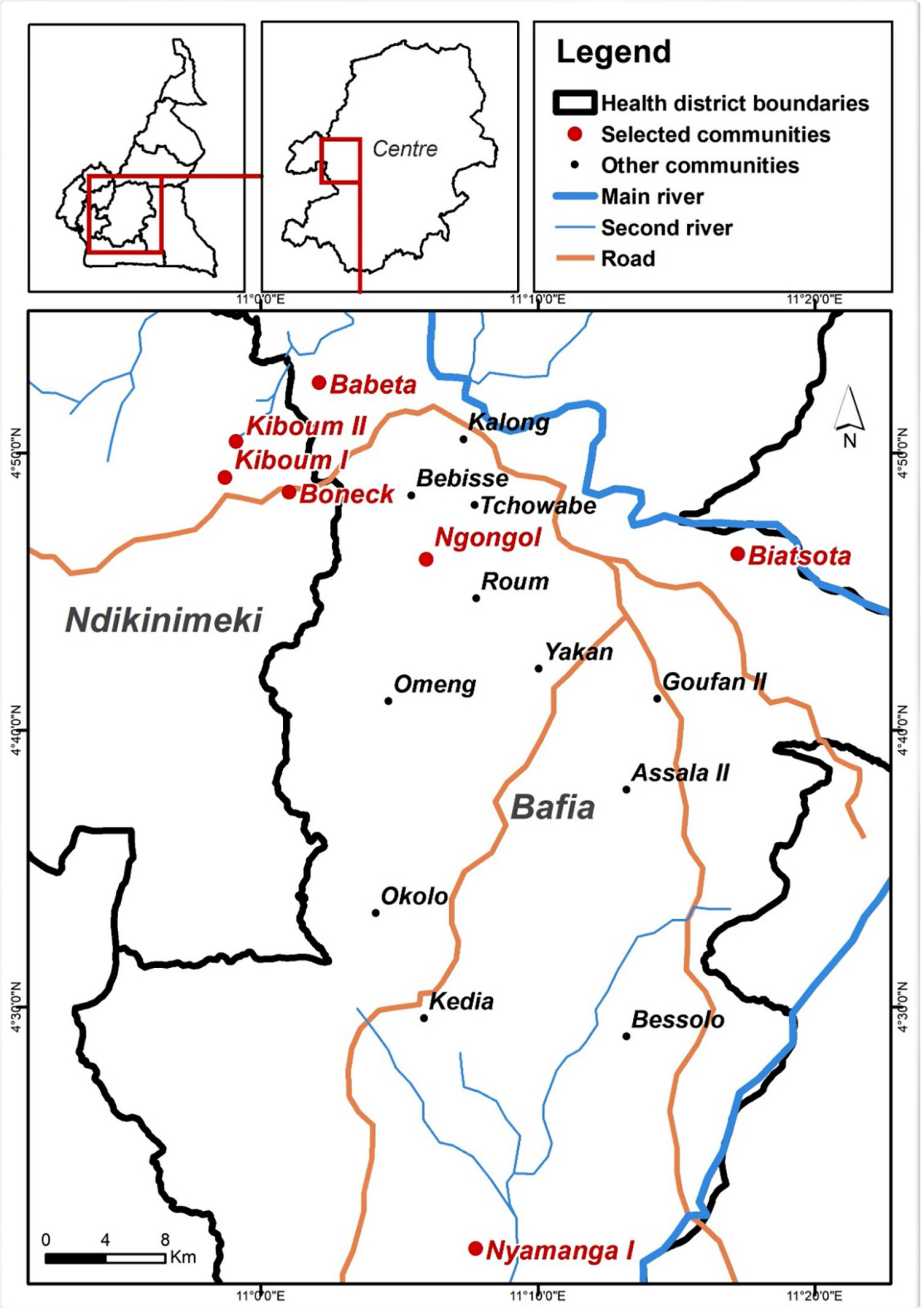
Map showing the study area with the surveyed communities. Red dots indicate the surveyed communities in the Bafia and Ndikinimeki health districts. The base layer map was sourced at OpenStreetMap (https://www.openstreetmap.org/#map=9/3.8780/11.3434).

This study was carried out in the Bafia (4°45′00″N, 11°14′00″E) and Ndikinimeki (3°58′15.32″N, 10°54′8.46″E) health districts, located in the Mbam valley in the Centre Region of Cameroon (Fig 1). The climate in the Mbam valley is equatorial and organized into four seasons: a long dry season (November-February), a short rainy season (March-June), a short dry season (June-August) and a long rainy season (August-November). The mean annual temperature is 22.4°C and the average annual rainfall is 1,440 mm [21]. It is a forest-savannah transition zone with altitude ranging from 1,100 to 1,300 m. This area is watered by a complex hydrographical network made up of fast flowing rivers including Sanaga and its tributaries, the Mbam, Noun, Inoubou, Makombé and Makénéné rivers. These rivers are bordered by forest galleries which, in addition to partially immerged rocks, continuously provide supports (branch of trees, leaves) suitable for blackflies’ breeding. Socio-economic activities are mainly centered on agriculture, sand mining and fishing. These activities performed closed to the rivers, expose inhabitants to permanent contact with blackflies, the vectors of onchocerciasis.

### Onchocerciasis and CDTI in the Mbam valley

Bafia and Ndikinimeki health districts are historical hyperendemic foci for onchocerciasis where transmission is ongoing/persistent despite more than two decades of annual ivermectin-based preventive chemotherapy [22,23]. Indeed, ivermectin mass chemotherapy started in the late 1990s, with significant improvement in treatment coverage since 2012 [23]. After more than two decades of preventive chemotherapy, the prevalence (nodule and microfilarial) and intensity of onchocerciasis infection significantly decreased to meso-endemic levels [23]. The last ivermectin treatment campaign in these two health districts was carried out in June 2019, prior to this survey that took place in February 2021 (that is 20 months apart).

### Study design

This study was conducted following a quantitative cross-sectional multi-stage stratified cluster sampling design. The first stage consisted in purposive selection of two health districts among the 32 constituting the Centre Region of Cameroon, based on their high endemicity prior to intervention and ongoing transmission of onchocerciasis. The second stage was a purposive selection of two health areas in each of the selected health districts, and the third stage was a purposive selection, in each of the selected health areas, of two communities with prior COVID-19 existing data. For comparability purpose, data collected prior to COVID-19 and cessation of preventive chemotherapy were retrieved from the most recent surveys conducted in the same communities using the same sampling strategy and diagnostic method. Community-based surveys were therefore carried out in seven communities of the Ndikinemiki and Bafia health districts (Fig 1). The minimal sample size was calculated based on the prevalence of onchocerciasis set at 50% with a precision of 5% and a confidence interval of 95%. Therefore, 301 participants were needed to estimate the true prevalence of onchocerciasis in studied districts.

In each community, point-of-contacts were established with the help of community health workers and local authorities to organize participants’ enrollment. Participants aged ≥5 years old, either males or females, permanent residents or who lived for at least five years in the community were eligible for this survey. Socio-demographic data (including age, gender and location), as well as compliance and history to ivermectin treatment (proportion of individuals eligible to ivermectin, that is those above 5 years old, who have ingested the drug during the last five years) were collected using a semi-structured questionnaire. After registration and brief interview, enrollees underwent clinical and parasitological examinations.

### Clinical examination to assess morbidity due to onchocerciasis

Clinical symptoms associated with onchocerciasis were sought in an isolated and well illuminated room. Clinical examinations consisted on (i) evaluating visual acuity, (ii) skin examination for skin lichenified, loss of skin elasticity, changes in skin pigmentation and skin itching; (iii) palpation of subcutaneous nodules, paying particular attention to bony prominences of the torso, iliac crests and upper trochanter of the femurs. Mobile masses beneath the skin, firm and painless were clinically identified as onchocercal nodules [24]. Clinical signs were registered as “Present” or “Absent”, and the number and location of identified onchocercal nodules were recorded.

### Parasitological examination to search for skin microfilaridermia

The parasitological diagnosis of onchocerciasis was established using the skin snip technique. Briefly, the skin was cleaned with antiseptic solution (ethanol 70%) and two skin biopsies (one from each posterior iliac crest) were collected using a sterile 2 mm corneoscleral Holth-type punch. Skin samples from each participant were introduced in two separate wells of a 96 well microtiter plate containing 100 μL of saline solution (0.9% NaCl). The microtiter plate was sealed with parafilm membrane to prevent potential spell-over and incubated for 24 hours at room temperature. After the incubation period, the fluid containing skin was analyzed and emerged *Onchocerca volvulus* microfilariae were counted using a microscope at low-magnification (100x) by trained laboratory technicians [25]. Individual microfilarial densities were expressed as the arithmetic mean number of microfilariae in the two skin snips (mf/ss) [26,27].

### Data analysis

Relevant data including socio-demographic information (age, gender, village of residence), compliance to CDTI, clinical data (nodules, itching frequency), microfilariae counts were recorded into a purpose-built Microsoft Access database and subsequently exported into R Studio (version 4.0.3) for statistical analyses. Categorial variable including presence of palpable nodules, skin lesions, skin microfilaridermia were expressed as the percentage of infected/affected individuals among the total number examined; the 95% confidence interval (CI) for proportion/prevalence was calculated using the exact method. Continuous variables including microfilarial densities and age were expressed as geometric mean associated to 95% Confidence Interval (95% CI). In addition to the skin microfilarial density, intensity of infection was measured by the community microfilarial load (CMFL) estimated as the geometric mean number of microfilariae per skin snip (mf/ss) among adults aged ≥20 years, using the log(x+1) transformation to take into account zero counts, especially after multiple rounds of ivermectin treatments.

Proportions/prevalence were compared between co-variates using Chi-square test (with Yates correction for continuity and Fisher exact test for small sample sizes). Data related to intensities of infection were compared among age and sex classes using Mann-Whitney and Kruskal-Wallis tests. The difference between the prevalence and intensity of infection prior to treatment interruption and that obtained from this assessment was assessed by the Chi-square test and the Mann Whitney test, respectively. For all statistical analyses, *p*-values were reported with threshold of significance set at 5%.

## Results

### Socio-demographic characteristics of the study participants

A total of 504 enrollees (225 from Ndikinimeki and 279 from Bafia health districts) were examined for onchocerciasis. The sex-ratio (M/F: 1.01) of enrollees was slightly male-biased (50.3% of participants were males), and their age ranged from 5 to 99 years old (Median: 38 years; IQR: 15–54). A total of 84.1% of study participants lived in the surveyed health districts from birth.

### History of ivermectin mass treatments

An overall of 81.8% (95% CI: 71.2–85.3%) of participants reported to have swallowed ivermectin at least once during the past 5 years (2015–2019) with no difference among sexes and health districts of origin of participants (S1 Table). The trend in ivermectin compliance increased with age, with significant difference between age classes (Chi-square = 12.94, df = 4, *p-value* = 0.01) (S1 Table).

The proportion of permanent compliers—participants who have reported taking ivermectin every year for the past five years—was estimated at 10.7% (95% CI: 8.3–13.9%), evenly distributed among sexes (Chi-square = 2.51, df = 1, *p-value* = 0.11), but significantly higher in the Ndikinimeki health district compared to Bafia health district (Chi-square = 40.9, df = 1, *p-value* < 0.0001). A significant difference in ivermectin permanent compliance was also found among the 7 villages (Chi-square = 78.19, df = 6, *p-value* < 0.0001) and Kiboum 1 exhibited the highest permanent compliance level (34.0%; 95% CI: 24.6–44.6) (S1 Table). The proportion of systematic non-compliers, defined as individuals who reported never having received ivermectin in the last five years, was estimated 17.6% (95% CI: 14.4–21.2%), and was evenly distributed among sexes (Chi-square = 0.01, df = 1, *p-value* = 0.84), health districts (Chi-square = 3.4, df = 1, *p-value* = 0.06) and village of residence of participants (Chi-square = 12.138, df = 6, *p-value* = 0.05). However, children under 10 years of age had the highest proportion of non-compliers to CDTI compared to other age groups (Chi-square = 44.5, df = 4, *p-value* < 0.0001).

### Clinical burden of onchocerciasis

Itching was the predominant onchocerciasis-associated symptoms reported by participants (60.6%; 95% CI: 56.1–64.9), and was significantly higher in Bafia health district compared to Ndikinimeki health district (Chi-square = 10.45, df = 1, *p-value* = 0.001) and among participants aged above 50 years old (Chi-square = 18.001, df = 4, *p-value* = 0.001). A total of 4.3% (95% CI: 2.7–6.5) of enrollees suffered from onchocercal rash and only four (0.8%; 95% CI: 0.2–2.0%) participants suffered from chronic onchodermatitis with “leopard skin” appearance. The overall prevalence of palpable nodules was 0.8% (95% CI: 0.2–2.0%); all participants with palpable nodules were above 40 years old and originating from the Bafia health district. The number of palpable nodules ranged from 1 and 2 per participant and were detected at the level of trochanter and scapula. An overall 2.3% (95% CI: 1.0–3.8) of participants were found with reduced visual acuity.

### Prevalence and intensity of infection

The overall microfilaridermia (mf) prevalence was 13.5% (95% CI: 10.6–16.7), and was similar between health districts district (Chi-square = 1.02, df = 1, *p-value* = 0.31) and among age groups (Chi-square = 2.43, df = 4, *p-value* = 0.655) (Table 1). All surveyed communities were classified as hypo-endemic based on the microfilaridermia prevalence (below 35%) according to standard classification of onchocerciasis level of endemicity [28]. The community Kiboum 2 belonging to Ndikinimeki health district displayed the highest prevalence (21.4%; 95 CI: 11.6–34.4) (Chi-square = 18.53, df = 6, *p-value* = 0.005), whereas Boneck belonging to the same district presented with the lowest mf prevalence (2.7%; 95 CI: 0.3–9.3) (Table 1). The mf prevalence was significantly higher in males compared to females (Chi-square = 7.305, df = 1, *p-value* = 0.007) (Table 2).

**Table 1:** Communities microfilarial prevalence and intensity in Bafia and Ndikinimeki health districts.

Health District	Village	Number examined	Mf Prevalence % (95% CI)	Mf density GM (95% CI)	CMFL (Mf/ss)
**Bafia**	Babeta	69	5.8 (1.6–14.1)	2.607 (0.61–11.21)	0.052
	Biatsota	50	20.0 (10.0–33.7)	2.315 (1.27–4.22)	0.200
	Ngongol	95	19.0 (11.6–28.2)	3.076 (1.63–5.81)	0.288
	Nyamanga 1	65	15.3 (7.6–26.4)	1.553 (1.63–2.82)	0.276
	***Total Bafia***	***279***	**15.05 (11.1–19.8)**	**2.405 (1.68–3.45)**	***/***
**Ndikinimeki**	Boneck	75	2.7 (0.3–9.3)	24.97 (6.94–89.86)	0.134
	Kiboum 1	94	12.8 (6.8–21.2)	2.649 (1.44–4.86)	0.231
	Kiboum 2	56	21.4 (11.6–34.4)	4.781 2.20–10.39)	0.611
	***Total Ndikinimeki***	***225***	***11*.*6 (7*.*7–16*.*5)***	**4.134 (2.49–6.86)**	***/***
**Total**		**504**	**12.4 (9.7–15.6)**	**2.958 (2.19–3.99)**	**0.240**

**GM:** Geometric mean; **CMFL**: Community Microfilarial Load; **Mf**: Microfiariae; **ss**: skin snip

**Table 2:** Sex and age-group microfilarial prevalence in Ndikinimeki and Bafia health districts.

Health District	Age group	Number examined		Number infected (%)		
		Male (n)	Female (n)	Male n (%)	Female n (%)	Overall
Bafia	[5–9]	14	24	5(35.7)	3(12.5)	21.0
	[10–14]	23	12	7(30.4)	1(0.0)	22.8
	**Children**	**37**	**36**	**12(32.4)**	**3(8.3)**	**20.5**
	[15–29]	20	25	3(15.0)	1(4.0)	8.9
	[30–49]	24	41	5(20.8)	6(14.6)	16.9
	>50	49	47	8(16.3)	3(6.4)	11.4
	**Adults**	**93**	**113**	**16(17.2)**	**10(8.8)**	**12.6**
Ndikinimeki	[5–9]	13	9	0(0.0)	1(11.1)	4.5
	[10–14]	14	13	1(7.1)	1(7.7)	7.4
	**Children**	**27**	**22**	**1(3.7)**	**2(9.1)**	**6.1**
	[15–29]	18	16	6(33.0)	3(18.7)	26.5
	[30–49]	36	30	6(16.7)	1(3.3)	10.6
	>50	42	34	4(9.5)	3(8.8)	9.2
	**Adults**	**96**	**80**	**16(16.7)**	**7(8.7)**	**13.1**
**Total**		**253**	**251**	**45(16.2)**	**22(8.8)**	**13.3**

The mean of microfilarial density of the overall studied population, estimated at 2.96 mf/ss (95% CI: 2.19–3.99), was unevenly distributed among communities with a significantly higher density in Kiboum 2 (Chi-square = 18.03, df = 6, *p-value* = 0.006) (Table 1) and sexes (Chi-square = 8.77, *p-value* = 0.003); no significant difference was found between age groups (Chi-square = 2.24, df = 4, *p-value* = 0.69). Considering the intensity of infection at the level of the communities surveyed, the CMFL ranged from 0.052 mf/ss in Babeta village (Bafia health district) to 0.611 mf/ss in Kiboum 2 (Ndikinimeki health district).

### Trends in prevalence and intensity of *O*. *volvulus* infection and impact of one-year CDTI disruption

**Fig 2:**
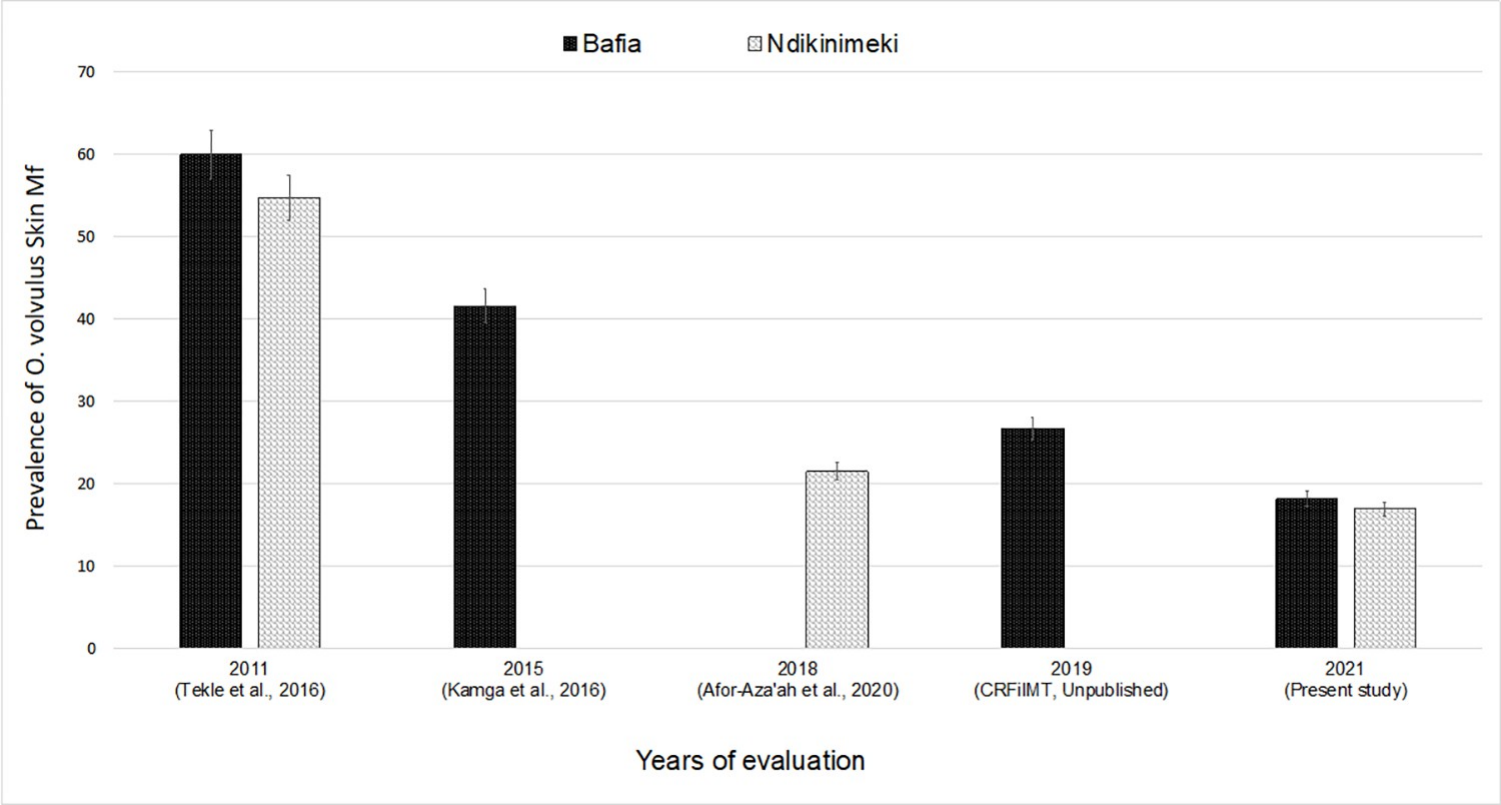
Trends in *Onchocerca volvulus* microfilarial prevalence over the last ten years (2011–2021) of CDTI in the Mbam valley. Dark bars represent the mf prevalence in the Bafia health district, and grey bars indicate the mf prevalence in the Ndikinimeki health district.

**Fig 3:**
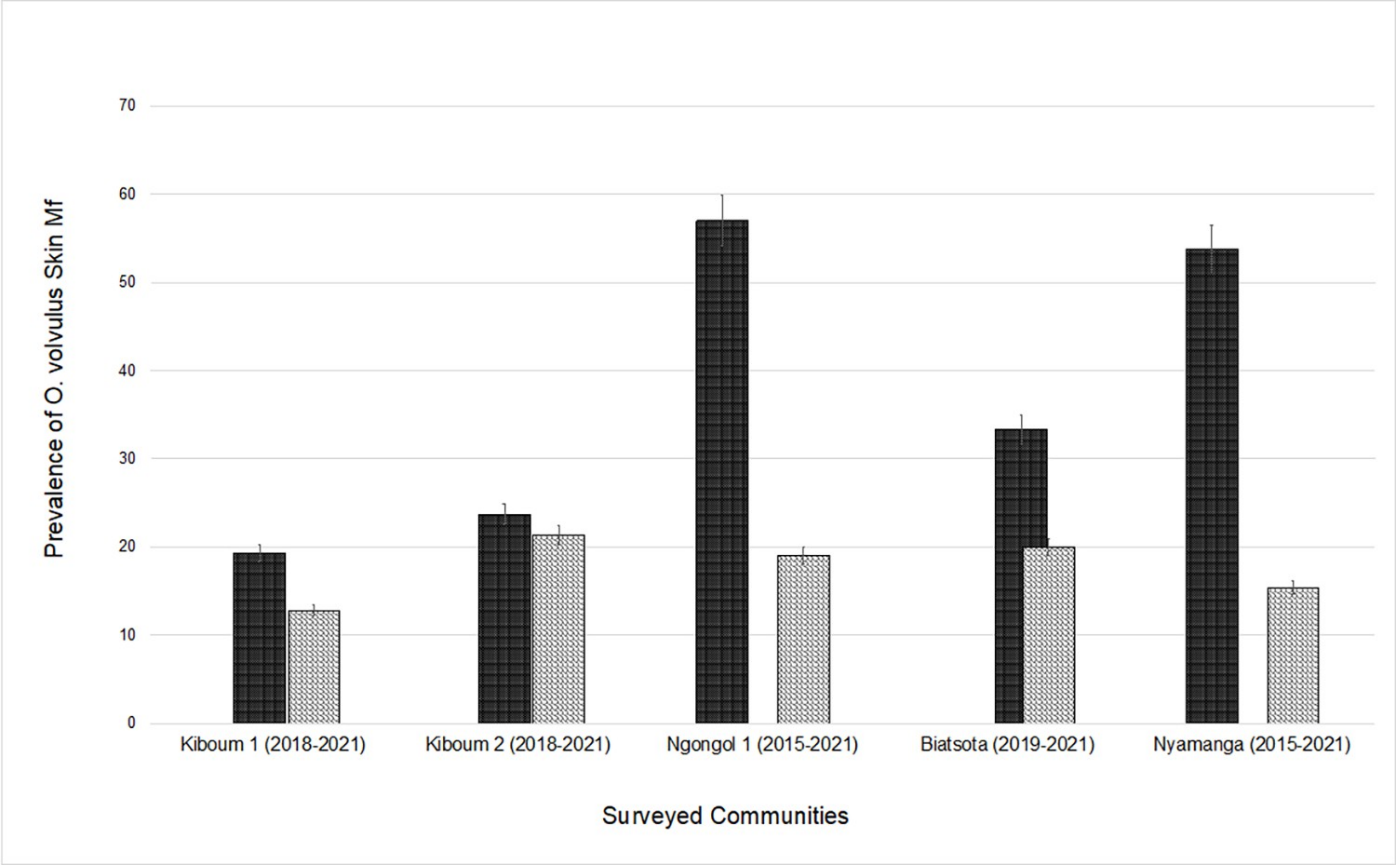
Trends in *Onchocerca volvulus* microfilarial prevalence over the last ten years (2011–2021) of CDTI in surveyed communities. Dark bars represent the prior COVID-19 mf prevalence, and grey bars represent the mf prevalence in the 2021 surveys.

Fig 2 displays the trends in mf prevalence in surveyed communities in the Ndikinimeki and Bafia health districts over the past 10 years. The trend in mf prevalence was similar between 2018 (pre-COVID-19 pandemic evaluation) and 2021 in the Ndikinimeki health district (Chi-square = -0.986, df = 1, *p-value* = 0.162), but significantly higher in 2019 (pre-COVID-19 pandemic evaluation) compared to 2021 in the Bafia health district (Chi-square = 22.35, df = 1, *p-value* < 0.0001) (Fig 2). The general mf prevalence decreasing trend was also observed at the community level after the latest survey in Biatsota (Bafia health district) where the prevalence dropped from 33.3% (95% CI: 25.1–38.0) to 20.0% (95% CI: 10.0–33.7) (Chi-square = 0.851, df = 1, *p-value* = 0.035); The same trend was observed in the other surveyed communities of the Bafia health district, including Nyamanga (Chi-square = 1.57, df = 1, *p-value* < 0.01) and Ngongol (Chi-square = 1.57, df = 1, *p-value* < 0.01). In the communities of Ndikinimeki health district, the decreasing trend of the prevalence was not significant either in Kiboum 1 where prevalence shifted from 19.3% (95% CI: 11.1–31.3) to 12.8% (95% CI: 6.8–21.2) (Chi-square = 1.57, df = 1, *p-value* = 0.057) or in Kiboum 2 where mf prevalence reduced from 23.7% (95% CI: 14.7–36.0) to 21.4% (95% CI: 11.4–34.4) (Chi-square = 0.05, df = 1, *p-value* = 0.814) (Fig 3).

Overall, the trends in the intensity of infection decreased in surveyed health districts since the previous evaluation despite one year of CDTI discontinuation, though the magnitude of the reduction in infection was health district dependent. Indeed, in the Bafia health district, the mean of the microfilarial densities decreased from 4.44 ± 14.24 mf/ss (mean ± SD) in 2019 to 0.37 ± 1.36 mf/ss in 2021 (current study) (U = 21410, *p-value* < 0.0001) and CMFL dropped from 1.08–1.33 mf/ss (lowest and highest CMFL recorded in 2019, respectively) to 0.052–0.288 mf/ss between 2019 and 2021. The same trend of decreasing microfilaridermia densities was noticed in Ndikinimeki health district which dropped from 1.607 ± 6.93 mf/ss (mean ± SD) in 2018 [22] to 1.15 ± 6.405 mf/ss in 2021 (current study) (U = 14366, *p-value* < 0.02). However, the CMFL remained generally stable between 2018 (0.383–0.538 mf/ss) and 2021 (0.134–0.611 mf/ss).

## Discussion

This study was carried out in a context where ivermectin-based MDA was disrupted because of COVID-19-related restrictions imposed by the WHO. Hence, at the date of the survey ivermectin had not been distributed for 20 months since the last treatment campaign, i.e. about 8 months delay if one considers that the next treatment would have taken place 12 months after the last round of ivermectin treatment. The objective of this study was to assess the burden of onchocerciasis in the Mbam Valley (Central Cameroon) and to compare the epidemiological trends following one year of CDTI discontinuation with the predictions of existing mathematical models.

Although data from earlier surveys in this area were not available, the present cross-sectional assessment of onchocerciasis-associated morbidity showed that itching was the predominant symptom reported, with over 60% of those surveyed suffering from pruritus even though mf prevalence was significantly lower than that. This high prevalence could be explained by the existence of other sources of skin itching in studied areas, including arthropod bites, scabies or other dermatologic conditions. Interestingly, the proportion of skin itching reported in Bafia health district was the highest among those living in less than 5 kilometers from the breeding sites (first line communities) with the highest exposure to blackfly bites (Fig 1).

The prevalence of palpable nodules estimated at 0.8% (95% CI: 0.2–2.01%), superimposed on previous assessments, indicates a steadily declining trend in onchocerciasis morbidity in both health districts since the beginning of mass treatment [22,23,29].

Longitudinal analysis of preceding studies based on *O*. *volvulus* mf prevalence, showed a constant decline in mf prevalence in surveyed health districts and communities, moving from an initial mf prevalence ≥ 60% (hyper-endemicity level) prior to the implementation of the CDTI in 1998 [30] to a mf prevalence < 35% (hypo-endemicity level) by 2021. Historically and based on the similar levels of mf prevalence in Bafia and Ndikinimeki health districts and their similar bioecology, socio-demographic features and timing of CDTI, there was a similar impact of annual CDTI in both Bafia (going from an mf prevalence of 92,8% in 1992 to 19% in 2021) and in Ndikinimeki (95.2% in 1992 to 21.4% in 2021). Despite the disruption of CDTI due to COVID-19, there was a continued downward trend in mf prevalence especially in Bafia health district. Indeed, the average mf prevalence in the surveyed communities was globally lower than those obtained during previous evaluations carried out in 2019, 2018 and 2015 in the communities of Biatsota, Kiboum (1 and 2) and Ngongol, respectively.

This observation confirms some of the scenarios released by mathematical models (ONCHOSIM) predicting the dynamism of mf prevalence consecutive to 1 and 2 years of CDTI interruption in endemic areas with long-term MDA [20]. The decreasing mf prevalence observed despite the disruption of the yearly round of CDTI, especially in Bafia health district, could be related to the biology of the parasite if the proportion of “older” infected individuals having received 15 or more rounds of MDA (whereby adult worms may no longer be able to produce mf) are significantly overrepresented in the population [31]. This postulate is supported by the evidence of calcified nodules identified during this study (100% of individuals with palpable nodules were negative to skin mf). In addition, the prepatent period of *O*. *volvulus* can be up to 2–3 years, making the diagnosis of potential cases of recent infections challenging.

The decreasing of CMFL in surveyed communities between the present study and previous data collected 2 years and 3 years ago in Bafia (Biatsota) and Ndikinimeki HD (Kiboum 1 and Kiboum 2), respectively, was unexpected since Mf densities were expected to increase within the communities. This observation can be explained—and is likely strengthened—by the low Mf prevalence within the population and the multiple previous annual rounds of CDTI. Indeed, the longstanding ivermectin pressure may certainly have had a significant or more prolonged embryostatic effect on female adult worms and increased mortality [32]. This assumption is supported by studies carried out in Latin America that showed that recurrent treatments with ivermectin significantly increased the proportion of dead and moribund adult female worms, as well as reduced the production of microfilariae by surviving females [33,34]. Even though the treatment regimen were different in the two contexts (annual in Africa vs bi- or multi-annual in Latin America), the cumulative effect of decades of CDTI could have led to similar effect.

## Conclusions

Disruption of one round of annual ivermectin-based chemoprevention exhibited very limited impact on prevalence and intensity of *Onchocerca volvulus* infection. These results are consistent with the mathematical model predictions (ONCHOSIM), indicating that additional efforts deployed by control program to catch up the gap of a very short-term disruption of ivermectin-based preventive chemotherapy (by applying one shot biannual then classical annual MDA) would not be cost-effective in settings with long histories of optimal and uninterrupted preventive chemotherapy. However, to accelerate transmission interruption in these settings where the infection seems to persist even though continuously declining, alternative/complementary treatment strategies [35] are urgently needed to reach the WHO 2030 goal [16].

## Supporting information

S1 STROBE Checklist STROBE checklist. (DOCX)

S1 Table Distribution of CDTI adherence and onchocerciasis morbidity by village, age group and sex of participants in the study districts. (DOCX)
