# Supplementary material for: Impact of short-term discontinuation of ivermectin-based chemoprevention on onchocerciasis transmission in endemic settings with long history of mass drug administration
Source: PLoS Negl Trop Dis. 2023 Apr 14;17(4):e0011250. doi: 10.1371/journal.pntd.0011250 (PMC10132555; doi:10.1371/journal.pntd.0011250)
Supplement: S1 Table — (DOCX) [file pntd.0011250.s002.docx]

**S1 Table**: Distribution of CDTI adherence and onchocerciasis morbidity by village, age group and sex of participants in the study districts

| Category | Variables | Number examined* | History of treatment | | Onchocerciasis Morbidity | | | | | |
| --- | --- | --- | --- | --- | --- | --- | --- | --- | --- | --- |
|  |  |  | **Sys non-compliers n (%)** | **Permanent compliers n (%)** | **Itching n (%)** | **Nodules n (%)** | **Leopard Skin n (%)** | **Onchocercal rash n (%)** | **Loss of Visual acuity n (%)** |  |
| Districts | Bafia | 279 | 42 (15.1) | 7 (2.5) | 186 (66.7) | 4 (1.4) | 2 (0.7) | 10 (3.6) | 8 (2.9) |  |
|  | Ndikinimeki | 226 | 47 (20.8) | 47 (20.8) | 117 (51.8) | 0 (0.0) | 2 0.9) | 11 (4.9) | 3 (1.3) |  |
|  | **Total** | **505** | **89 (17.6)** | **54 (10.7)** | **303 (60.0)** | **4 (0.8)** | **4 (0.8)** | **21 (4.2)** | **11 (2.2)** |  |
|  | ***p-value*** |  | **0.11** | **0.0001*** | **0.001*** | **/** | **/** | **/** | **/** |  |
|  |  |  |  |  |  |  |  |  |  |  |
| Villages | Babeta | 69 | 6 (8.7) | 1 (1.4) | 47 (68.1) | 0 (0.0) | 0 (0.0) | 3 (4.3) | 2 (2.9) |  |
|  | Biatsota | 50 | 13 (26.0) | 0 (0.0) | 29 (58.0) | 1 (2.0) | 0 (0.0) | 1 (2.0) | 2 (4.0) |  |
|  | Ngongol | 95 | 14 (14.7) | 6 (6.3) | 67 (70.5) | 2 (2.1) | 0 (0.0) | 3 (3.2) | 2 (2.1) |  |
|  | Nyamanga | 65 | 9 (13.8) | 0 (0.0) | 43 (66.2) | 1 (1.5) | 2 (3.1) | 3 (4.6) | 2 (3.1) |  |
|  | Kiboum 1 | 94 | 19 (20.2) | 32 (34.0) | 41 (43.6) | 0 (0.0) | 1 (1.1) | 4 (4.3) | 0 (0.0) |  |
|  | Kiboum 2 | 57 | 8 (14.0) | 3 (5.3) | 36 (63.2) | 0 (0.0) | 0 (0.0) | 2 (3.5) | 0 (0.0) |  |
|  | Boneck | 75 | 20 (26.7) | 12 (16.0) | 40 (53.3) | 0 (0.0) | 1 (1.3) | 5 (6.7) | 3 (4.0) |  |
|  | ***p-value*** |  | **0.04*** | **< 0.0001*** | **0.003*** |  |  |  |  |  |
|  |  |  |  |  |  |  |  |  |  |  |
| Age Groups | [5-9] | 60 | 28 (46.7) | 1 (1.7) | 22 (36.7) | 0 (0.0) | 0 (0.0) | 6 (10.0) | 3 (5.0) |  |
|  | [10-14] | 62 | 4 (6.5) | 5 (8.1) | 40 (64.5) | 0 (0.0) | 0 (0.0) | 1 (1.6) | 0 (0.0) |  |
|  | **Children** | **122** | **32 (26.2)** | **6 (4.9)** | **62 (50.8)** | **0 (0.0)** | **0 (0.0)** | **7 (5.7)** | **3 (2.5)** |  |
|  | [15-29] | 79 | 18 (22.8) | 4 (5.1) | 45 (57.0) | 0 (0.0) | 0 (0.0) | 2 (2.5) | 3 (3.8) |  |
|  | [30-49] | 132 | 15 (11.4) | 18 (13.6) | 82 (62.1) | 0 (0.0) | 1 (0.8) | 9 (6.8) | 2 (1.5) |  |
|  | ≥50 | 172 | 24 (14.0) | 26 (15.1) | 114 (66.3) | 4 (2.3) | 3 (1.7) | 3 (1.7) | 3 (1.7) |  |
|  | **Adults** | **383** | **57 (14.9)** | **48 (12.5)** | **241 (62.9)** | **4 (1.0)** | **4 (1.0)** | **14 (3.7)** | **8 (2.1)** |  |
|  | ***p-value*** |  | **< 0.0001** | **0.01*** | **0.0015*** |  |  |  |  |  |
|  |  |  |  |  |  |  |  |  |  |  |
| Sex | Female | 252 | 46 (18.3) | 21 (8.3) | 155 (61.5) | 3 (1.2) | 3 (1.2) | 12 (4.8) | 5 (2.0) |  |
|  | Male | 253 | 43 (17.0) | 33 (13.0) | 148 (58.5) | 1 (0.4) | 1 (0.4) | 9 (3.6) | 6 (2.4) |  |
|  | ***p-value*** |  | **0.84** | **0.1** | **0.5** | **/** | **/** | **/** | **/** |  |

** Children under 5 were not interviewed during the survey since they are excluded from CDTI.*
